# Real-Time Polymerase chain reaction trends in COVID-19 patients

**DOI:** 10.12669/pjms.37.1.3000

**Published:** 2021

**Authors:** Sana Abbas, Aisha Rafique, Beenish Abbas, Rashid Iqbal

**Affiliations:** 1 Dr. Sana Abbas, MCPS. National University of Medical Sciences Rawalpindi, Pakistan; 2 Dr. Aisha Rafique, MBBS. National University of Medical Sciences Rawalpindi, Pakistan; 3 Dr. Beenish Abbas, FCPS. Foundation University, Rawalpindi, Pakistan; 4 Dr. Rashid Iqbal, FCPS Anaesthesia, FCPS Pain Medicine. National University of Medical Sciences Rawalpindi, Pakistan

**Keywords:** Coronavirus, Cough, Comorbid, PCR

## Abstract

**Objective:**

To assess trends of real-time Polymerase Chain Reaction test in Coronavirus infected Patients.

**Methodology:**

This cross-sectional analytical study was conducted at Tertiary Care Institute, Rawalpindi from March 2020 to June 2020. All patients confirmed COVID positive by real-time Polymerase Chain Reaction (PCR) with recent travel history, close contact with known diagnosed patients and had symptoms of fever or upper respiratory tract with body aches. Nasopharyngeal swabs were taken and results generated within 48 hours. Positive PCR was admission criteria follow up was carried out at 7^th^ and 8^th^ day, with negative PCR were discharged. However, those who had persistent positive PCR on the 8th day were tested again on 11th and 12^th^ day. Those with persistent positive results beyond 12^th^ day were shifted to specialized quarantine centres.

**Results:**

A total of three hundred and ninety-two patients with mild to moderate illness, PCR positive for COVID 19 were included study with age range 9 - 45 and mean 33.22±7.98 years. A total of 8 (2%) patients were females and 384(98%) males. The duration of the negative test result was Mean ± Std. Deviation 9.05±2.00 with 7 – 8 days 152(38.8%)in and 11 – 12 days in 160(40.8%). PCR results on Day 7 and 8 were negative in 144(36.7%) patients whereas positive in 248(63.3%). PCR results on Day 11 and 12 were negative in 312(79.6%) patients whereas positive in 80 (20.4%).

**Conclusion:**

To conclude Real-Time Polymerase Chain Reaction (rT-PCR) inclines to give false negative results additionally can stay positive in asymptomatic patients for moderately longer-term. Hence decision to discharge ought to be intricately adjusted between RT-PCR, clinical judgement, radiological examinations, and biochemical assays.

## INTRODUCTION

The coronavirus pandemic (COVID-19), initiated in December 2019, is currently established. Due to its fast spread and rising number of infected cases, rapid and accurate detection of the virus is ever more pivotal. It will aid to control the sources of infection. Effective detection will prevent the illness progression.^1^ COVID-19 is a severe acute respiratory infection which carried high mortality is associated with comorbidities. Real-time reverse transcription-Polymerase Chain Reaction is currently the most reliable diagnostic method worldwide.^2^ Guidelines for diagnostic workup endure to grow as awareness of COVID-19 improves and convenience of testing facilities intensify.^3^

PCR process includes amplification of well-defined DNA segment. It is multiplied thousands of times. This amplification renders this DNA enough to be identified. Viruses such as SARS-CoV-2 contain RNA.^4^ Respiratory sample is collected from the person being tested. It is treated with certain chemicals. These break down extraneous substances. It allows the RNA to be removed from the sample and analyze.^5^ Reverse transcription-polymerase chain-reaction uses reverse transcription to adapt the extracted RNA into DNA. It then uses PCR to amplify a piece of the resulting DNA. It causes doubling of the target region with each cycle. Creating enough to be examined to determine if it matches the genetic code of SARS-CoV-2.^6^ A fluorescent signal is created when amplification occurs. Once the signal reaches a threshold, the test result is considered positive. If no viral sequence is present, amplification will not occur. Thus resulting in a negative PCR.^7^

Real-time PCR (RT -PCR) provides advantages during the PCR portion of this process. It enables high-Output and more dependable instrumentation. It has become the preferred method.^8^ Altogether, the joint technique has been designated as real-time RT-PCR or quantitative RT-PCR.^9^ Respiratory samples obtained by various methods, including a nasopharyngeal swab or sputum sample, as well as on saliva can be used as sample.^10^

Our study was based on the fact that Polymerase Chain Reaction (RT-PCR) can be positive in asymptomatic patients and can remain positive for an extended duration in patients who recovered from symptomatic illness, therefore, will constantly be a threat for disease transmission as a carrier.

## METHODS

This cross-sectional analytical study was conducted at Tertiary Care Institute, Rawalpindi from March 2020 to May 2020 approval was taken from the ethical research committee of the Institute (ERC Number –243/ERC).

The minimum sample size required for this cross-sectional study was 246, calculated by using formula (n = [deff *np(1-p)]/ [(d2/z21-α/2*(n-1)+p*(1-p)]-open epi calculator), with 95% confidence level and 5% margin of error where the hypothesized sensitivity of Polymerase Chain Reaction Test in Coronavirus disease was considered to be 66%-80% (80%±5 ) as reported by Struts.^11^ A non-probability consecutive sampling methodology was employed and total (n = 392) participants were enrolled.

A total of Three hundred and Ninety-Two patient were included in the study to eliminate bias and aid to a reliable analysis. Informed consent was taken and patients were briefly described the purpose of the study. Ethical approval was taken from the hospital ethical review committee. All patients were COVID positive by real-time Polymerase Chain Reaction (PCR). Testing was carried out only in those individuals who had recent travel history, close contact with known diagnosed patients and had symptoms of fever or upper respiratory tract with body aches. Patient with comorbid like Diabetes mellitus, Hypertension, Ischemic Heart Disease, Asthma and Rheumatologic disease were also included. Patients with a diagnosis of pneumonia due to other causes, tuberculosis, interstitial lung, chronic kidney disease, immunosuppression disease and chronic obstructive pulmonary disease were excluded. Nasopharyngeal swabs were taken from all patients and assessed by real-time PCR. Reports were generated within 48 hours. PCR was taken by trained laboratory assistant wearing personal protective equipment. Patient was immediately admitted after the first PCR was positive.

Patients with fever were advised HCQ 400 mg twice a day for two days followed by 200 mg twice daily for another four days. Tablet azithromycin 500 mg once daily and tablet ivermectin once daily was given for five days. Treatment was further aided with vitamin C, calcium and zinc supplements. Battery of tests carried out included C – reactive Protein, Liver Function tests, Chest X-ray, Serum Ferritin, Blood Complete count and coagulation profile as part of management.

Indoor patients were tested on 7th day, if negative PCR result obtained subsequent test on 8th day was performed, negative result was criteria for discharge. However, those who had persistent positive PCR were tested again after three days that is 11^th^ and 12^th^ day. Two consecutive negative reports were considered criteria for discharging the patient. However, patients who remained positive even on 12^th^ day results they were shifted to specialized quarantine centres to ensure overcrowding prevention and further transmission. Their tests were performed on a weekly interval and consequently patients were discharged upon receipt of two consecutive negative test reports. Follow up of patients after discharge was carried out in the outdoor unit.

**Table–I:** 8^th^ and 12^th^-day Polymerase Chain Reaction Test Results.

		*Polymerase Chain Reaction Test 8^th^ Day*		*P-value*

		Negative	Positive	
Polymerase Chain Reaction Test 12^th^ Day	Negative	144(100.0%)	168(67.7%)	<.001 *
	Positive	-	80(32.3%)	

* Significant p-value; p-value was calculated by applying Chi-square test

**Table-II:** Polymerase Chain Reaction Results in Patients with Comorbid.

		*Comorbid*			*P-value*

		HTN	DM	Asthma	
Polymerase Chain Reaction	Negative	40(100.0%)	16(66.7%)	8(100.0%)	<.001*
Test 12^th^ Day	Positive	-	8(33.3%)	-	
Polymerase Chain Reaction	Negative	32(80.0%)	8(33.3%)	-	<.001*
Test 8^th^ Day	Positive	8(20.0%)	16(66.7%)	8(100.0%)	

* Significant p-value; p-value was calculated by applying Chi-Square Test

Data was entered and analysed by using data management software IBM SPSS (version 23.0). The descriptive statistics of continuous variables were presented as mean and standard deviation, while categorical data frequencies and percentages were used. Categorical grouped data were analyzed by Chi-square Test. A p-value of ≤0.05 was considered to be statistically significant.

## RESULTS

Three hundred ninety two patients were enrolled in the study with age range 9 - 45 and mean 33.22±7.98 years. A total of eight (2%) patients were females and 384(98%) males. Comorbid prevalence was hypertension in 40 (10.2%), diabetes mellitus in 24(6.1%), and asthma in 8(2.0%) patients. Serum C-reactive protein (CRP) was elevated in 48 (12.2%) upon admission, with a range of 7.00 - 10.00 and mean value 8.50±.96, however, returned to normal in 392(100%) patients by Day – 8 of admission. 392 (100%) patients were completely asymptomatic with resolution of consolidation upon chest x-ray by 12^th^ day of hospital admission. Duration of negative test result was Mean ± Std. Deviation 9.05±2.00 with 7 – 8 days 152(38.8%)in and 11 – 12 days in 160(40.8%). PCR results on 7^th^ and 8^th^ day were negative in 144(36.7%) patients whereas on 11th and 12^th^ day were negative in 312(79.6%) patients.

## DISCUSSION

Review of our analysis elaborated that (100%) patients were asymptomatic with resolution of consolidation upon chest x-ray by the twelfth day of indoor admission. Duration of negative test outcome was mean ± standard deviation 9.05±2.00.152(38.8%) patients were negative by eighth day and 160(40.8%) patients upon twelfth-day results. PCR results on Day 11 and 12 were positive in 80 (20.4%) patients. Research examination is compatible with the various published studies on the subject.

**Table-III:** Correlation of Polymerase Chain Reaction Test Results with Cough, Fever & Chest X-ray.

		*8^th^ Day Polymerase Chain Reaction Test*		*p-value*

		Negative	Positive	
Fever and Cough	No	112(77.8%)	232(93.5%)	<.001*
	Yes	32(22.2%)	16(6.5%)	
CXR	NAD	112(77.8%)	232(93.5%)	<.001*
	Bilateral Patches	32(22.2%)	16(6.5%)	

* Significant p-value; p-value was calculated by applying Chi-Square Test

**Table-IV:** Correlation of Comorbid with Duration of Positive PCR.

		*Duration*			*p-value*

		Persistently Positive	7^th^ Day	11^th^ Day	
Comorbid	HTN	-	32(80.0%)	8(33.3%)	<.001*
	DM	8(100.0%)	8(20.0%)	8(33.3%)	
	Asthma	-	-	8(33.3%)	

* Significant p-value; p-value was calculated by applying Chi-Square Test

Lan et al. reported persistent positive RT-PCR test results in patients recovered from mild to moderate coronavirus disease. Four patients had two successive negative RT-PCR test results with hospitalization duration of 12 to 32 days. PCR elaborated negative test results in five to thirteen days, therefore discharged with standard quarantine protocols. After four to five days time span positive test result was encountered again despite of asymptomatic course of disease. No contact history or contamination of relatives was established. On the other hand among our patients results remained positive for a longer period.^12^

Xie et al specified Chest CT relationship to negative RT-PCR testing in COVID-19 pandemic. One hundred sixty seven patients were enrolled and 5 (3%) patients presented with positive chest CT although PCR was negative. Thus all patients were assumed to be infected with coronavirus disease therefore quarantined, with repeat test of PCR swab.155 patients (93%), had concordant positive test results for RT-PCR and CT Scan. None of our patient presented with positive chest x-ray and negative PCR results. However consolidation upon chest x-ray completely resolved by 12^th^ day in most of the patients with negative PCR test results.^13^

Xiao et al completed an illustrative report at a unique profile of RT-PCR in 301 COVID-19 patients in China. 85 (28.2%) patients still tested positive for PCR at last follow up. The positive rate of RT-PCR was observed most frequent from day 0−7 (97.9%), with a decline to 68.8% (day 8−14), 36.3% (day 15−21), 30.0% (day 22−28) and 26.3% (28 days). These findings were consistent with our analysis as PCR results remained positive in 80 (20.4%)patients beyond 12^th^ day. 74 tests (37 sets) were acquired from both throat swabs and nasal swabs simultaneously. Throat swabs specimens concluded false negative in 41.3% patients.^14^

Bwire et al composed a letter to the editorial manager in which they explained trends observed globally. Data from Germany revealed that two out of 114 explorers (1.8%) were concluded false negative after PCR test results. Japanese residents had been determined to have the contamination after at first testing negative, attempted PCR twice. Followed by a positive test result on 12^th^ day of suspicion. A distributed report on clinical attributes of 138 hospitalized patients with COVID-19 in Wuhan, China, archived that fever was available in 98.6% (136/138) affirmed fever as a symptom of illness in hospitalized patients, whereas two (1.4%) didn’t present with fever. Temperature probably won’t be a sufficient screening tool, as it can drop travellers incubating the illness and add to the importation of the infection. At present, RT-PCR is an authentic test in recognizing both indicative and asymptomatic COVID-19.^15^

Yaun et al elucidated that PCR Assays turned Positive in 25 discharged patients. Mean age was of 28 years with 17 females and six children. Past clinical records, enumerated disease duration as 15.36±3.81 and treatment with ritonavir/lopinavir and IFN-α, comparable with other discharged patients. Before discharge, chest tomography (CT) enhancements and two successive negative outcomes (24 hours of interim) of PCR test. Discharged patients were followed up with cloacal swab and nasopharyngeal swabs every three days.14 patients tested positive for Cloacal swab tests and 11 patients indicated test results of nasopharyngeal swab test. Therefore, these 25 patients had a mean time frame of 7.32±3.86 from last negative to positive again.^16^

Long et al diagnosed COVID-19 infectivity with PCR or CT Scan. Thirty-six cases were diagnosed. 35 patients had CT findings consistent with disease whereas one patient exhibited normal CT Scan. Among 30 patients PCR was positive whereas six patients had negative test results. Therefore, CT was conclusive in 97.2% however PCR had diagnostic predictability of 84.6%.^17^

Tahamtan et al in their article inferred that the PCR tends to negative outcome with upper-respiratory-tract tests, recommended trial of lower respiratory tract samples analysis if feasible. RT-PCR, CT scan and clinical manifestations could eliminate the likelihood of false-negative results.^18^

Hence our study emphasizes on unsual and variable pattern of Polymerase Chain Reaction (RT-PCR) among patients which is crucial as disease duration, carrier state and incubation period can not be decided with certainty. Patients can have positive test results despite of being asymptomatic or can be encountered for an extended duration in patients who recovered from symptomatic illness, therefore, will constantly be a threat for disease transmission as a carrier. Adequate spacing strategy among indoor patients has association with early achievement of negative PCR. This aspect has substantial impact of disease spread in poorly resourced and over populated third world countries.

### Limitations of the study

This Pandemic has shown an expeditious course and since our study was based on limited period therefore considering these findings preliminary more research work is to be carried out and conceptualized to evaluate the disease process.

## CONCLUSION

Real-Time Polymerase Chain Reaction (rT-PCR) inclines to give false negative results additionally can stay positive in asymptomatic patients for moderately longer-term. Hence decision to discharge ought to be intricately adjusted between RT-PCR, clinical judgement, radiological examinations, and biochemical assays.

### Authors’ Contribution:

**SA:** Conceived, designed and did statistical analysis & editing of manuscript.

**AR, BA & RI:** Did data collection and manuscript writing.
